# Endophthalmitis nach Bindehautperforation eines Glaukom-Gelstentimplantates

**DOI:** 10.1007/s00347-020-01077-7

**Published:** 2020-02-28

**Authors:** Laila El Moussaoui, Yassin Djalali-Talab, Peter Walter, Niklas Plange, David Kuerten, Matthias Fuest

**Affiliations:** Klinik für Augenheilkunde, Uniklinik RWTH Aachen, Pauwelsstr. 30, 52074 Aachen, Deutschland

**Keywords:** Glaukom, Chirurgie, Minimal-invasiv, Endophthalmitis, Implantat, Abiotrophia defectiva, Glaucoma, Surgery, Minimally invasive, Endophthalmitis, Implant, Abiotrophia defectiva

## Abstract

Intraokulare Infektionen durch *Abiotrophia defectiva *sind sehr selten. Hier berichten wir von einer 57-jährigen Patientin, welche sich 3 Monate nach erfolgter komplikationsloser Kataraktoperation mit Implantation eines Glaukom-Gelstents am rechten Auge mit einer *Abiotrophia-defectiva-*assoziierten Endophthalmitis bei uns vorstellte. Die Patientin klagte zuvor über eine Rötung im Bereich der nasal oberen Bindehaut des rechten Auges sowie über Schmerzen 2 Wochen vor Auftreten der Endophthalmitis. Eine 2‑wöchige topische Steroidtherapie ohne Antibiotikaschutz brachte eine kurzfristige Besserung. Die Patientin stellte sich nun bei uns mit einem Hypopyon, einer akuten Visusverschlechterung und starken periokulären Schmerzen seit dem frühen Morgen vor. Der Gelstent hatte spontan die Konjunktiva perforiert. Es erfolgte die unmittelbare Therapie mit lokaler und systemischer Antibiose. Sechs Stunden danach wurde eine Pars-plana-Vitrektomie mit intraokularer Gabe von Antibiotika durchgeführt. Durch eine zeitnahe Therapie konnte in diesem Fall ein relativ benigner Verlauf erreicht werden. Im klinischen Alltag sollte bei Patienten, die sich nach glaukomchirurgischen Eingriffen mit akuter Visusverschlechterung und Schmerzen präsentieren, dringend an eine mögliche spontane Bindehautperforation und Late-onset-Endophthalmitis gedacht werden. Zudem ist zu empfehlen, dass eine unklare Konjunktivitis nach Glaukomchirurgie immer antibiotisch abgedeckt und engmaschiger kontrolliert werden sollte.

## Abstract (en)

Intraocular infections associated with *Abiotrophia defectiva* are rare. This article reports the case of a 57-year-old woman with endophthalmitis associated with *Abiotrophia defectiva* 3 months after uncomplicated cataract surgery combined with the implantation of a glaucoma gel-stent in the right eye. The patient had complained of redness of the right upper nasal conjunctiva and pain for 2 weeks prior to the endophthalmitis. A topical steroid eyedrop treatment without antibiotic additives had temporarily improved the situation. The patient presented with hypopyon, acute deterioration of vision and severe periocular pain of the right eye since the early morning. The gel-stent had spontaneously perforated the conjunctiva. The patient was immediately started on local and systemic antibiotics and underwent pars plana vitrectomy with intravitreal antibiotic application 6 h after presentation. Unlike other ocular infections with *Abiotrophia defectiva*, this case had a relatively benign course most likely due to the prompt intervention. In clinical routine, patients, who present with acute deterioration of vision and pain after glaucoma surgery, should be examined urgently considering a possible spontaneous conjunctival perforation and late onset endophthalmitis. Additionally, conjunctivitis of unclear origin following ocular surgery should always be treated with antibiotics, particularly when steroids are administered and monitored closely.

## Anamnese

Eine 57-jährige Patientin mit primärem Offenwinkelglaukom hatte Ende 2018 eine kombinierte, unkomplizierte Kataraktoperation mit Implantation eines Glaukom-Gelstents (XEN®45 Gel Stent, Allergan, Dublin, Irland) mit 0,1 ml Mitomycin C 0,02 % subkonjunktival am rechten Auge bei uns erhalten. Die Indikation hierfür wurde wegen eines insuffizienten Augeninnendruckes am rechten Auge bei einer Unverträglichkeit auf multiple lokale drucksenkende Augentropfen gestellt. An beiden Augen wurde bei guter Verträglichkeit zuletzt Timolol hydrogenmaleat 5 mg/ml unkonserviert appliziert. Vor dem geplanten Eingriff lag der bestkorrigierte Visus rechts bei beginnender Kataraktbildung bei 0,8 und links bei 1,0, der intraokulare Druck war rechts 25 mm Hg und links 18 mm Hg. Der intraoperative und frühe postoperative Verlauf gestaltete sich komplikationslos.

Die letzte elektive Vorstellung der Patientin erfolgte 1,5 Monate nach der Gelstentimplantation. Der Visus mit eigener Korrektur war beidseits 1,0 und der Augeninnendruck rechts 12 mm Hg und links 15 mm Hg. Am rechten Auge bestanden eine reizfreie Bindehaut und eine klare Hornhaut. Weiterhin zeigte sich ein prominentes und reizfreies Filterkissen im Bereich der nasal oberen Bindehaut. Avaskuläre Areale oder größere Zysten waren nicht zu erkennen. Der Gelstent lag gut positioniert im Kammerwinkel und ragte ca. 1 mm in die Vorderkammer sowie ca. 2 mm frei beweglich unter die Bindehaut. Intraokular bestand kein Reiz. Drei Monate nach erfolgtem Eingriff stellte sich die Patientin notfallmäßig mit seit dem Morgen bestehenden starken periokulären Schmerzen und einer Visusverschlechterung des rechten Auges auf 0,2 mit eigener Korrektur vor. Anamnestisch berichtete die Patientin, seit 3 Wochen eine Rötung im Bereich der nasal oberen Bindehaut des rechten Auges festgestellt zu haben. Sie habe daraufhin einen niedergelassenen Augenarzt konsultiert. Dieser habe aufgrund einer Entzündung für 2 Wochen topische Steroide verordnet. Eine antibiotische Therapie habe sie nicht erhalten. Anamnestisch wird bis zur notfallmäßigen Vorstellung zunächst über eine initiale Besserung des Befundes im Sinne einer rückläufigen Bindehauthyperämie berichtet.

## Klinische Befunde und Diagnose

**Figure 1:**
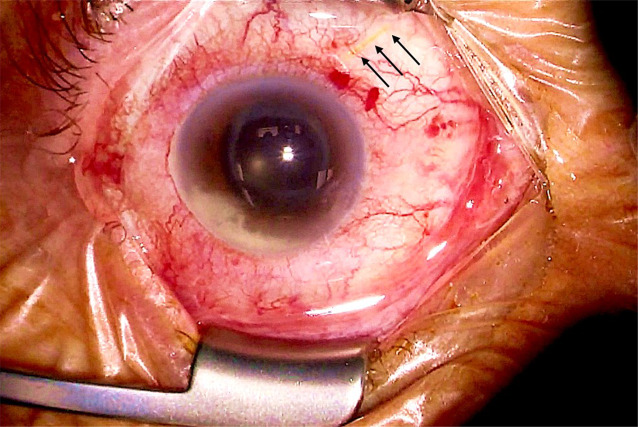

Bei Vorstellung lag der Fernvisus mit bester Korrektur rechts bei 0,2 und links bei 1,0. Der Augeninnendruck war rechts 33 mm Hg und links 12 mm Hg. Im Bereich der Bindehaut des rechten Auges zeigte sich nasal oben bei 2 Uhr ein Gelstent, welcher die stark injizierte Bindehaut perforiert hatte und ca. 2–3 mm freilag (Abb. 1 und 2a). Zudem bestand ein deutlicher Vorderkammerreiz mit Hypopyon und Fibrin (Abb. 1). Der Fundus war durch eine starke Glaskörperverdichtung nur schemenhaft einsehbar. Größere Infiltrate oder Blutungen waren aber nicht zu erkennen. In der Ultraschalluntersuchung lag die Netzhaut zirkulär an und der Glaskörper wies deutliche Verdichtungen auf.

**Figure 2:**
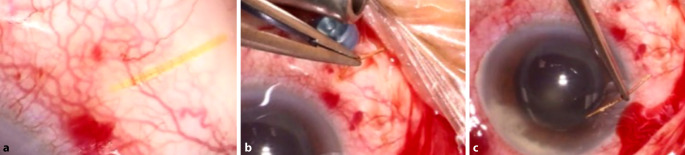

## Therapie und klinischer Verlauf

**Figure 3:**
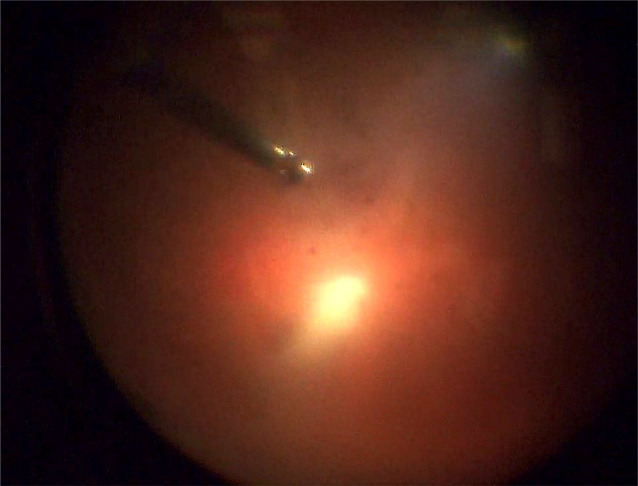

Bei Verdacht auf Endophthalmitis bei perforiertem Gelstent erfolgten eine notfallmäßige stationäre Aufnahme und Indikationsstellung zur umgehenden Pars-plana-Vitrektomie. Es wurden eine sofortige Lokaltherapie mit Moxifloxacinhydrochlorid-Augentopfen viertelstündlich sowie eine intravenöse Systemtherapie mit Vancomycin 2‑mal 1 g und Ceftazidim 3‑mal 2 g täglich begonnen. Sechs Stunden nach initialer Vorstellung erfolgte am rechten Auge die 23-G-Pars-plana-Vitrektomie mit Stentexplantation, Entnahme von Vorderkammer- und Glaskörperproben, Luftfüllung, Applikation von Vancomycin 1 mg/0,1 ml, Ceftazidim 2 mg/0,1 ml und Dexamethason 0,4 mg/0,1 ml in den Glaskörperraum. Der Gelstent ließ sich ohne jeden Widerstand aus der Sklera herausziehen (Abb. 2b, c). Intraoperativ war der Funduseinblick wegen der dichten Glaskörperinfiltration zunächst schlecht. Nach ausgiebiger Vitrektomie zeigte sich ein nichtischämischer Fundus mit wenigen Fleckblutungen (Abb. 3).

Postoperativ erhielt die Patientin zusätzlich zur systemischen Antibiose am rechten Auge lokal Moxifloxacinhydrochlorid-Augentropfen stündlich, Cyclopentolathydrochlorid-Augentropfen 2‑mal täglich und Timololhydrogenmaleat 5 mg/ml-Augentropfen 2‑mal täglich sowie Prednisolonacetat-Augentropfen 1 % stündlich. Die Sehschärfe lag am Entlassungstag (fünfter postoperativer Tag) rechts bei Handbewegungen und der Augeninnendruck bei 16 mm Hg. Der Glaskörperraum war ca. zu zwei Drittel mit Luft gefüllt. Der Augeninnendruck war im gesamten stationären Aufenthalt stets in einem normotonen Bereich.

Nach Rückmeldung des Mikrobiologen 16 h nach der Operation zeigten sich im Direktpräparat grampositive Kokken. Da der genaue Erreger noch unklar war, wurde die systemische Therapie mit Vancomycin und Ceftazidim zunächst fortgesetzt. Als Entlassungstherapie wurde die intravenöse Antibiose auf Cefuroxim 500 mg 2‑mal täglich oralisiert.

Die bakteriologische Kultur ergab 9 Tage nach der Vitrektomie* Abiotrophia defectiva* in der Vorderkammer- und in der Glaskörperprobe. Im Antibiogramm erwies sich dieser Keim als multisensibel. Die mikrobiologische Untersuchung mittels PCR (Polymerase-Kettenreaktion) der eingesendeten Proben bestätigte den Befund. Der Gelstent selbst blieb ohne Keimnachweis.

**Figure 4:**
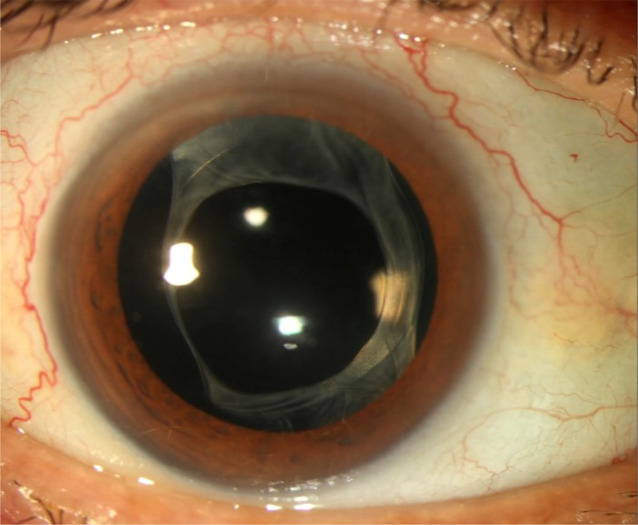

**Figure 5:**
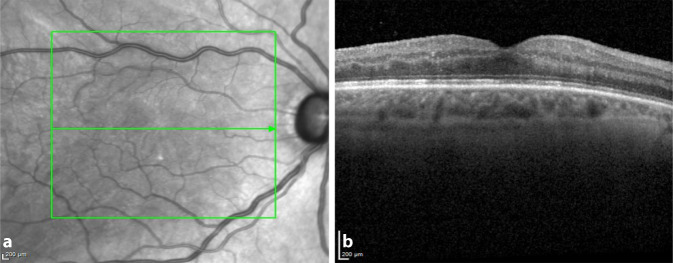

Bei der Verlaufskontrolle nach einer Woche konnten rechts eine bestkorrigierte Sehschärfe von 0,1 und ein Augeninnendruck von 17 mm Hg gemessen werden. Der vordere Augenabschnitt und der Glaskörperraum waren reizarm mit nur vereinzelten Zellen. Die Netzhaut wies keine größeren Blutungen auf, es waren lediglich vereinzelte Fleckblutungen erkennbar. Rechts wurden Prednisolonacetat 1 %- und Moxifloxacinhydrochlorid-Augentropfen auf 5‑mal täglich reduziert, während Timololhydrogenmaleat 5 mg/ml-Augentropfen 2‑mal täglich beidseits fortgesetzt und die systemische Antibiose abgesetzt wurde. Drei Wochen postoperativ besserte sich der bestkorrigierte Visus rechts auf 0,2, der Augeninnendruck betrug 22 mm Hg, sodass die antiglaukomatöse Therapie um Clonidinhydrochlorid 0,54 mg/0,5 ml 3‑mal täglich ergänzt wurde. Der vordere Augenabschnitt war reizfrei (Abb. 4). Im Makula-OCT konnte kein Makulaödem nachgewiesen werden, die Makulastruktur war noch aufgelockert (Abb. 5). Es erfolgte eine Umstellung von Prednisolonacetat 1 %- auf Fluorometholon-Augentropfen 3‑mal täglich. Dieses sollte wöchentlich um 1 Tropfen reduziert und schließlich abgesetzt werden.

Eine Verlaufskontrolle nach 3 Monaten ergab rechts einen bestkorrigierten Visus von 0,4 und links 1,0. Der Augeninnendruck lag unter Therapie mit Timolol hydrogenmaleat 5 mg/ml-Augentropfen 2‑mal täglich beidseits und rechts zusätzlich Clonidin hydrochlorid 0,54 mg/0,5 ml 3‑mal täglich bei beidseits 16 mm Hg. Im vorderen Augenabschnitt des rechten Auges zeigte sich die Bindehaut weitestgehend reizarm, intraokular bestand kein Reiz. Die Netzhaut war unauffällig.

## Diagnose

Endophthalmitis durch *Abiotrophia defectiva* nach spontaner Bindehautperforation eines Glaukom-Gelstents.

## Diskussion

Dies ist die erste Fallbeschreibung einer Endophthalmitis nach Spontanperforation eines Glaukom-Gelstents, verursacht durch *Abiotrophia defectiva.*

Die postoperative Endophthalmitis ist eine der gefürchtetsten Komplikationen eines intraokularen Eingriffs. Die häufigsten Ursachen für eine exogene Endophthalmitis sind eine vorausgegangene intravitreale Medikamenteninjektion oder Kataraktoperation. Die Kardinalsymptome bestehen aus einer plötzlichen Visusverschlechterung und Schmerzen. Klinisch zeichnet sich eine Endophthalmitis klassischerweise durch einen Vorderkammerreiz mit Hypopyon sowie Glaskörperinfiltration mit reduziertem oder fehlendem Funduseinblick aus [1].

Die Diagnosestellung erfolgt klinisch. Die Probengewinnung aus der Vorderkammer und dem Glaskörper dient dem Keimnachweis, um eine möglichst gezielte und erregerspezifische Therapie durchzuführen. Es ist aber zu berücksichtigen, dass eine negative mikrobiologische Untersuchung eine Endophthalmitis nicht ausschließt. Etwa 20–30 % der Fälle bleiben ohne Keimnachweis [1]. Der therapeutische Standard ist die intravitreale Gabe einer Antibiose nach erfolgter Vorderkammerspülung und Vitrektomie [1].

Die Visusprognose ist stark abhängig von folgenden Faktoren: Visus bei Vorstellung, Zeitpunkt der therapeutischen Intervention und als Hauptprognosefaktor, welcher Keim verantwortlich ist. Infektionen mit *Staphylokokken *und *Propionibacterium acnes* haben eine bessere Prognose, während Infektionen mit* Streptokokken, Bacillus spp., Enterokokken, Pseudomonas* und *Aspergillus spp.* eine schlechte Prognose haben [1].

In der Literatur wird für das Auftreten einer Endophthalmitis nach Kataraktoperation eine Inzidenz zwischen 0,03 und 0,3 % angegeben. In den meisten Fällen, ca. 75 %, tritt die Endophthalmitis in den ersten postoperativen Wochen auf. Als häufigste Erreger bei der akuten Endophthalmitis nach Kataraktoperation konnten koagulasenegative Streptokokken ermittelt werden, welche Bestandteil der normalen Bindehautflora sind. Bei der verzögert auftretenden Endophthalmitis dagegen konnten Studien *Propionibacterium acnes* oder Pilze als Haupterreger nachweisen [1].

Nach Sickerkissenchirurgie tritt eine Endophthalmitis sehr selten akut binnen 4 Wochen auf. Häufiger wurde eine Endophthalmitis nach 1,5 bis 7 Jahren beobachtet. Als Hauptrisikofaktor gilt eine dünne Sickerkissenwand. Die Inzidenz für eine Endophthalmitis nach Trabekulektomie liegt bei <2 % [2]. Als weitere Risikofaktoren werden die Sickerkisseninfektion, Myopie, Blepharitis, Langzeitgebrauch topischer Medikamente sowie eine dünne Bindehaut und in diesem Sinne ein höheres Lebensalter angegeben [2, 3]. Bei der akuten Form finden sich am häufigsten koagulasenegative Staphylokokken, in einem Drittel der Fälle *Streptococcus viridans* und bei der spät auftretenden Form Streptokokken und gramnegative Keime. Die Prognose ist bei Verläufen mit Enterkokken oder Streptokokken ungünstiger [1].

Die mikroinvasive Glaukomchirurgie („minimally-invasive glaucoma surgery“ [MIGS]) ist eine innovative Therapieoption in der Behandlung des mild bis moderat fortgeschrittenen Glaukoms.

Der XEN® 45 Stent ist das erste Ab-interno-MIGS-Implantat, welches eine subkonjunktivale Filtration ermöglicht. Dieser hydrophile zylindrische Schlauch besteht aus nicht degradierbarer Schweinegelatine und vernetztem Glutaraldehyd [4]. Das Lumen hat einen Durchmesser von 45 μm, die Länge beträgt 6 mm [4]. Durch seine materielle Zusammensetzung ist der Gelstent so flexibel, dass er sich dem Widerstand der Konjunktiva hingibt, sodass das Perforationsrisiko der Konjunktiva theoretisch minimal ist [4]. Im Vergleich zur Trabekulektomie zeigen sich nach Implantation des Gelstents durch die geringere Invasivität weniger postoperative Komplikationen und eine schnellere postoperative Wundheilung [5, 6].

In der Literatur werden bislang nur wenige Fälle von Endophthalmitis nach Glaukom-Gelstentimplantation beschrieben. In 2 Fällen wird von einer Perforation der Konjunktiva durch den Gelstent berichtet. Es handelte sich um eine 89 Jahre alte Patientin, welche sich 5 Tage nach Glaukom-Gelstentimplantation vorstellte. Es werden eine Konservierungsmittelunverträglichkeit sowie eine jahrelange antiglaukomatöse Medikamentenanamnese angegeben [3]. Im zweiten Fall wird über einen 80-jährigen männlichen Patienten berichtet, bei dem 4 Monate nach Glaukom-Gelstentimplantation eine Endophthalmitis bei Perforation der Konjunktiva diagnostiziert wurde [7]. In den beiden Fällen aus der Literatur kann das hohe Alter der Patienten, welches mit einer dünneren Konjunktiva einhergeht, als Risikofaktor für eine Perforation des Gelstent gesehen werden.

Anamnestisch bestand aber auch eine langjährige Therapie mit topischen Medikamenten in Kombination mit einer Tropfunverträglichkeit, was zu einer ausgedünnten Bindehaut führen kann. Bei unserer Patientin lag intraoperativ eine völlig intakte altersentsprechende Konjunktiva vor.

Risikofaktor für eine Endophthalmitis nach Glaukom-Gelstentimplantation scheint darüber hinaus der Gebrauch von MMC, welches den Heilungsprozess durch Ausdünnung des Bindehautepithels und -stromas verzögern kann [3], zu sein. Bei den geringen Fallzahlen von Endophthalmitiden nach Glaukom-Gelstentoperation ist die Bewertung von Risikofaktoren zum jetzigen Zeitpunkt jedoch schwierig.

In der Literatur werden leicht variierende MMC-Konzentrationen bei der Implantation von Gelstents angewendet. Meist werden 0,1 ml einer 0,01 oder 0,02 %igen MMC-Konzentration intraoperativ subkonjunktival gespritzt [3]. Unsere Patientin erhielt 0,1 ml MMC 0,02 % subkonjunktival.

Bei dünnerer Bindehaut sollte ggf. der Einsatz geringerer MMC-Konzentrationen oder 5‑Fluoruracil als Antimetabolit diskutiert werden.

Diskussionswürdig ist zudem eine Subtenonimplantation des Stents, um perioperative Mikrotraumata der Konjunktiva auszuschließen. Eine aktuelle Studie berichtet, dass bei einer Intra- und Subtenonlage des Gelstents, die als gleichwertig aufgeführt werden, niedrigere intraokulare Drücke im Vergleich zur Gruppe mit einem subkonjunktival gelegenen Stent ermittelt werden konnten [6]. Außerdem war die Revisionsrate mittels Needling bei einer Intra- und Subtenonlage des Gelstents geringer als bei einer subkonjunktivalen Lage [6]. Allerdings kann es eine Herausforderung sein, den Gelstent intraoperativ geplant sicher in eine Subtenon- oder Intratenonlage zu positionieren.

Ob eine intraoperative Perforation der Bindehaut ein Risikofaktor für eine spätere Endophthalmitis sein kann, ist unklar. Bei unserer Patientin war dies jedoch nicht der Fall.

In den meisten bisher publizierten Glaukom-Gelstent-assoziierten Endophthalmitisfällen wurde kein Keim nachgewiesen, lediglich in 1 Fall *Staphylococcus epidermidis* und in einem *Enterococcus faecalis *[3, 7].

*Abiotrophia defectiva, *der Keim, den wir in der Vorderkammer- und Glaskörperprobe unserer Patientin nachweisen konnten, findet sich kaum in der ophthalmologischen Fachliteratur. In den 1960er-Jahren ging man davon aus, dass NVS („nutritionally variant streptococci“) eine mutierte Form von *Streptococcus-viridans*-Stämmen sind [8]. Heute teilt man NVS in *Abiotrophia defectiva* und *Granulicatella adiacens, Granulicatella balaenopterae* und *Granulicatella elegans* ein [8, 9]. NVS finden sich in der Mundhöhle sowie im oberen Respirationstrakt, im Urogenital- und Gastrointestinaltrakt [8]. Okuläre Infektionen mit *Abiotrophia defectiva* sind äußerst selten. In der Literatur wird von 2 Fällen in England, 1 Fall in Deutschland und 2 Fällen in Spanien berichtet, in denen *Abiotrophia defectiva* als Keim identifiziert wurde [8, 9]. In den Fällen aus Deutschland und England traten die Symptome 3 bis 6 Tage nach erfolgter Kataraktchirurgie auf [8, 9].

In der Literatur werden die Verläufe einer *Abiotrophia defectiva*-induzierten Endophthalmitis als fulminant und schwerwiegend beschrieben [8]. Die Virulenz dieses Bakteriums wird v. a. bei der bakteriellen Endokarditis deutlich, wo der Keim häufiger (5 % der Fälle) als okulär vorkommt [10]. Hier konnten eine höhere Komplikations- und Mortalitätsrate und ein schlechteres Ansprechen auf antimikrobielle Therapien im Vergleich zu anderen Streptokokken bei der Endokarditis festgestellt werden [10]. Der klinische Verlauf war im vorliegenden Fall relativ benigne, was a. e. auf die kurze Latenzzeit bis zur Vitrektomie zurückzuführen ist.

Zusammenfassend lässt sich sagen, dass besonders peri-, aber auch bei den postoperativen Kontrollen nach Glaukom-Gelstentimplantation auf Schäden in der Konjunktiva geachtet werden muss. Insbesondere bei konjunktivalen Reizzuständen, muss das Filterkissen nach erfolgter Glaukom-Gelstentoperation ausgiebig untersucht werden. Im klinischen Alltag sollte bei Patienten, die sich mit Schmerzen und akuter Visusverschlechterung präsentieren und vor Monaten oder Jahren einen glaukomchirurgischen Eingriff bekommen haben, dringend an eine mögliche spontane Bindehautperforation und Late-onset-Endophthalmitis gedacht werden. Zudem ist zu empfehlen, dass eine unklare Konjunktivitis nach Glaukomchirurgie immer antibiotisch abgedeckt werden sollte und engmaschig kontrolliert werden muss.

## Fazit für die Klinik

- Bei Patienten mit plötzlich auftretenden Beschwerden nach Glaukom-Gelstentimplantation sollten Bindehaut und Filterkissen besonders genau inspiziert werden.
- Auch mehrere Wochen nach Implantation eines Glaukom-Gelstents kann es zu einer spontanen Bindehautperforation und Endophthalmitis kommen.
- Bei Anzeichen einer Entzündung des Filterkissens oder freiliegendem Stent sollte eine unverzügliche Überweisung in ein spezialisiertes Zentrum und bis dahin eine antibiotische Abdeckung erfolgen.

## References

[1] Durand ML (2017). Bacterial and fungal endophthalmitis. Clin Microbiol Rev.

[2] Jampel HD, Solus JF, Tracey PA, Gilbert DL, Loyd TL, Jefferys JL (2012). Outcomes and bleb-related complications of trabeculectomy. Ophthalmology.

[3] Karri B, Gupta C, Mathews D (2018). Endophthalmitis following XEN stent exposure. J Glaucoma.

[4] Richter GM, Coleman AL (2016). Minimally invasive glaucoma surgery: current status and future prospects. Clin Ophthalmol.

[5] Galal A, Bilgic A, Eltanamly R, Osman A (2017). XEN glaucoma implant with mitomycin C 1-year follow-up: result and complications. J Ophthalmol.

[6] Lenzhofer M, Strohmaier C, Sperl P, Hohensinn M, Hitzl W, Steiner V (2019). Effect of the outer stent position on efficacy after minimally invasive transscleral glaucoma gel stent implantation. Acta Ophthalmol.

[7] Olgun A, Imamoglu S, Karapapak M, Duzgun E, Kacar H (2018). Endophthalmitis after XEN gel stent implantation: 2 cases. J Glaucoma.

[8] Horstkotte MA, Dobinsky S, Rohde H, Knobloch JK, Hassenstein A, Kalitzky M (2010). Abiotrophia defectiva endophthalmitis with retinal involvement and infiltrative keratitis: case report and review of the literature. Eur J Clin Microbiol Infect Dis.

[9] Namdari H, Kintner K, Jackson BA, Namdari S, Hughes JL, Peairs RR (1999). Abiotrophia species as a cause of endophthalmitis following cataract extraction. J Clin Microbiol.

[10] Woo PC, Fung AM, Lau SK, Chan BY, Chiu SK, Teng JL (2003). Granulicatella adiacens and Abiotrophia defectiva bacteraemia characterized by 16S rRNA gene sequencing. J Med Microbiol.

